# Contributions from Long-Term Memory Explain Superior Visual Working Memory Performance with Meaningful Objects

**DOI:** 10.1101/2025.07.07.663569

**Published:** 2025-07-10

**Authors:** Hyung-Bum Park, Edward Awh

**Affiliations:** Institute for Mind and Biology, University of Chicago; Department of Psychology, University of Chicago

## Abstract

Visual working memory (WM) capacity has recently been claimed to be higher for meaningful objects compared to simple visual features, possibly due to richer and more distinctive representations. However, prior demonstrations of this advantage have typically compared performance with meaningful stimuli that are trial-unique to performance with a small set of repeated simple stimuli (e.g., colors). This design creates a confound between the strength of proactive interference (PI) and meaningfulness, such that PI is minimized for meaningful items compared to colors. Thus, improved WM performance with meaningful objects could reflect enhanced contributions from episodic long term memory (LTM), a memory system that is highly vulnerable to PI, rather than an increase in WM capacity. To examine this issue, Experiment 1 measured WM capacity for repeated colors, repeated meaningful objects, and trial-unique meaningful objects. We replicated the previously observed advantage for trial-unique objects over colors. Critically, this advantage was eliminated entirely with repeated meaningful objects that equated PI across the meaningful and simple stimuli, suggesting that minimal PI, not meaningfulness, drove this behavioral effect. In line with this hypothesis, hierarchical Bayesian dual-process signal detection modeling suggested that the advantage for trial-unique objects was due to enhanced familiarity-based LTM signals rather than recollection-based WM processes. To directly measure online storage in WM, Experiment 2 measured contralateral delay activity (CDA), an electrophysiological marker of the number of items stored in working memory. Although we saw the typical performance benefits for trial-unique objects over repeated colors, CDA activity across increasing set sizes revealed a common plateau for trial-unique meaningful objects and repeated colors, indicating a WM storage limit that is independent of stimulus meaningfulness. Thus, past demonstrations of superior memory performance with meaningful stimuli can be explained by a task design that minimized PI for meaningful compared to simple stimuli. When PI is equated, WM storage limits for simple and meaningful stimuli are equivalent.

Visual working memory (WM) temporarily maintains visual information to support goal-directed behavior (Cowan, 2001; Luck & Vogel, 2013). This online memory system appears to be highly limited in capacity. A broad range of studies has shown that WM-guided performance exhibits a sharp drop with memory displays containing more than three or four items (Adam et al., 2017; Alvarez & Cavanagh, 2004; Awh et al., 2007; Luck & Vogel, 1997; Ngiam et al., 2023; Park et al., 2017; van den Berg et al., 2014; Xie & Zhang, 2017; Zhang & Luck, 2008; Zhao et al., 2023). While some models argue against any strict limit on the number of items that can be stored (e.g., Bays, 2015; Oberauer & Lin, 2017; Schurgin et al., 2020; van den Berg et al., 2012, 2014), virtually all leading models converge on the view that only three to four items can be stored with *sufficient fidelity* to guide meaningful behaviors (e.g., Adam et al., 2017; Oberauer & Lin, 2017; Park & Zhang, 2024; Schurgin et al., 2020).

Critically, limits on visual WM performance appear to be primarily determined by the number of individuated items or “chunks”, rather than by the total amount of information contained within each chunk. Luck and Vogel (1997) originally demonstrated equivalent capacity estimates for single-feature and multi-feature objects, leading them to argue for object-based limits on visual WM capacity. Later research has shown a modest reduction in accuracy with multi-featured objects, yet these studies still demonstrate reliable “object-based benefits” wherein multiple features can be stored more effectively if bound together into a single object (Hardman & Cowan, 2015; Ngiam et al., 2024; Olson & Jiang 2002). Thus, visual WM capacity appears fundamentally item-based, even though multi-featured items are susceptible to probabilistic feature loss.

These object-based benefits typically arise from perceptual grouping (e.g., colored shapes), but chunks can also be formed on the basis of associative knowledge in long-term memory (LTM). For example, when observers learn reliable word-pair associations, WM performance for those pairs matches that for single words (Chen & Cowan, 2009). Likewise, consistently paired colors yield performance comparable to single colors (Brady et al., 2009). Critically, these associative benefits depend on explicitly retrievable LTM representations (Ngiam et al., 2019) and typically involve the time-consuming retrieval of chunk sub-components (Huang & Awh, 2018). Thus, superior performance with associative learning involves a close collaboration between WM and LTM. Importantly, the benefits of associative learning can be fully explained by more efficient chunking rather than an increase in the number of individuated items that can be stored in WM.

However, an important challenge to this fixed capacity perspective comes from recent reports of superior visual WM performance for meaningful, real-world objects compared to simple visual features (Asp et al., 2021; Brady et al., 2016; Chung et al., 2024; Torres et al., 2024). This “meaningful object benefit” has been attributed to enhanced semantic knowledge in LTM that promotes deeper encoding and more distinctive representations, potentially increasing the number of meaningful items concurrently stored in WM (Brady & Alvarez, 2011; Curby et al., 2009).

A natural alternative hypothesis, on the other hand, is that the extant behavioral demonstrations of increased WM storage for meaningful objects may instead reflect increased contributions from episodic LTM rather than increased WM storage. Critically, past demonstrations of the meaningful object benefit have employed *trial-unique* stimulus presentations, wherein each meaningful object was presented only once throughout the experiment. Performance in this task was then compared to performance with a small set of repeated feature values (e.g., eight categorically distinct colors^1^). This design inherently confounds the effects of meaningfulness with proactive interference (PI), the disruption of accessing new memories because of interference from similar past memories (Jonides & Nee, 2006; Kane & Engle, 2000; Park, 2025). Because PI is virtually absent with trial-unique stimuli, participants may effectively leverage global familiarity signals from LTM to improve memory performance (Standing, 1973). Consequently, performance in visual WM tasks can far exceed typical capacity limits when trial-unique stimuli are used, with some studies reporting capacity exceeding dozens of items (Endress & Potter, 2014). These findings suggest that enhanced performance with meaningful objects presented in a trial-unique manner may reflect contributions from LTM-based familiarity instead of true increases in WM storage.

Experiment 1 tested this hypothesis directly by comparing WM performance under conditions that either matched or minimized PI across three types of stimuli: trial-repeated colors, trial-repeated meaningful objects, and trial-unique meaningful objects. If the advantage for meaningful stimuli reflects semantic enrichment that enhances WM capacity, performance advantages should persist even when PI is matched. Conversely, if performance gains are due to episodic LTM retrieval enabled by reduced PI, then the meaningfulness advantage should disappear under equal interference. Moreover, formal modeling of familiarity and recollection components of performance was conducted to identify the specific aspect of memory that was enhanced by the use of trial-unique meaningful objects.

Another important approach to determine whether more meaningful objects can be stored in WM is to examine neural signatures of active maintenance. The contralateral delay activity (CDA) is an electroencephalogram (EEG) signature of WM storage that is defined by increased negative voltages in parietal-occipital electrodes contralateral to the stored items (Luria et al., 2016; Vogel & Machizawa, 2004). Critically, the CDA tracks storage in an *item-based* fashion, such that CDA amplitude is determined by the *number* of items stored, not the total amount of information associated with each item. For example, Woodman and Vogel (2008) showed equivalent CDA amplitudes for four simple orientations and four color-orientation conjunctions, despite the latter stimuli containing twice as many features. Thus, the CDA provides a powerful testbed for examining whether a larger number of meaningful objects can be actively maintained in WM.

Indeed, recent studies have reported larger CDA amplitudes for meaningful objects compared to simple features, particularly at higher memory loads (Asp et al., 2021; Brady et al., 2016; Chung et al., 2024; Thibeault et al., 2024; Torres et al., 2024; but see Quirk et al., 2020; Xie & Zhang, 2018). These findings have been offered as evidence for increased WM storage with meaningful objects. However, there is a critical problem with this interpretation, in that these studies employed only a single set size for measuring CDA activity^2^. Unfortunately, this design is not able to distinguish between contralateral negativities that are due to WM storage, and those that are due to low-level stimulus-driven effects. Woodman and Vogel (2008) reported one example of such a stimulus-driven effect with a study that measured CDA amplitudes for colors, orientation, and conjunction stimuli with set sizes of 2 or 4 items (Figure 1). Although CDA amplitudes were higher for orientations and conjunctions compared to color stimuli, this effect was *additive* with the number of items to be stored. Thus, while attending orientation yielded a larger contralateral negativity, this effect appears to be independent of changes in the number of items stored in WM. Indeed, WM performance was numerically superior for colors relative to orientation and conjunctions, further undermining the interpretation that more items were stored in the latter conditions. Also note that the Woodman and Vogel (2008) findings rule out models that predict CDA amplitude based on the total “information load”, because CDA amplitude was equivalent between orientation and conjunction stimuli, despite a doubling of the information load in the conjunction condition. Thus, the most parsimonious explanation of the main effect of stimulus type on CDA amplitude is that attention to specific kinds of visual features elicits an increased contralateral negativity that is independent of changes in WM storage.

Drew and colleagues (2011) provided another clear example of a stimulus-driven change in CDA amplitude that is independent of changes in WM storage. They observed increased CDA amplitudes during a multiple-object tracking task relative to a static WM task, despite equivalent numbers of attended items. Critically, this increased negativity in the moving object condition was *additive* with the number of items that were attended, arguing against any change in the number of items selected. Thus, when observers track moving objects, a contralateral negative wave is observed (in the same electrodes used to measure the CDA) that is distinct and dissociable from the neural signatures of WM storage.

The key point for the present discussion is that it is essential to measure CDA activity across multiple set sizes to distinguish between stimulus-driven effects that are additive with load, and true changes in WM storage. To this end, Experiment 2 examined CDA amplitudes with trial-repeated simple colors and trial-unique meaningful objects at three separate set sizes (1, 3, and 5 items). If the behavioral advantage observed with meaningful objects is due to an increased number of items stored in WM, then the function relating CDA activity to set size should diverge across stimulus types, yielding an interaction between stimulus type and set size. Specifically, WM storage of a larger number of meaningful items should result in a CDA by set size function that reaches an inflection at a larger set size than for simple colors. By contrast, if superior performance with meaningful objects is due to enhanced contributions from LTM, no difference in the shape of the CDA by set size function should be observed.

To anticipate the results, Experiment 1 replicated the behavioral advantage for trial-unique meaningful objects over repeated simple colors. This advantage, however, was eliminated when PI was matched between meaningful and simple stimuli. In addition, hierarchical Bayesian dual-process signal detection (DPSD) modeling indicated that the behavioral advantage with trial-unique meaningful objects was solely accounted for by familiarity-based retrieval rather than recollective memory access. Experiment 2 again replicated the behavioral benefit for trial-unique meaningful objects over repeated colors, yet the shape of the CDA by set size function was equivalent across stimulus types. Collectively, these results indicate that online storage in WM is not enhanced with meaningful stimuli, though they do afford strong performance benefits when PI is minimized (Endress & Potter, 2014).

## Experiment 1

To test whether superior visual WM performance for meaningful objects reflects enhanced WM capacity or reduced PI, Experiment 1 independently manipulated meaningfulness and PI. We compared recognition performance across three stimulus conditions: repeated colors, repeated meaningful objects, and trial-unique meaningful objects. If the meaningful object benefit reflects a genuine expansion of WM capacity, it should persist regardless of stimulus repetition. Conversely, if the benefit is due to the absence of PI with trial-unique stimuli, the advantage should disappear in the repeated meaningful object condition that equates PI. To further evaluate contributions of WM and episodic LTM, we analyzed participants’ confidence judgments using hierarchical Bayesian DPSD modeling, allowing separate estimates of recollection (context-bound WM) and familiarity (episodic LTM) signals.

### Method

#### Participants

Thirty-one volunteers (18 female) participated in the experiments and received monetary compensation ($20 per hour). Participants were aged between 18 and 31 years (*M* = 22.3, *SD* = 3.2), reported normal or corrected-to-normal visual acuity, and provided informed consent according to procedures approved by the University of Chicago Institutional Review Board.

Sample size was determined via an a priori power analysis (G*Power 3.1; Faul et al., 2009), targeting 85% power to detect a medium effect size (Cohen’s *d* > 0.5) in primary comparisons between stimulus conditions at an alpha level of 0.05, based on prior studies investigating visual WM capacity differences between meaningful objects and color stimuli (Brady et al., 2016; Chung et al., 2023, 2024).

#### Stimuli and Procedure

Stimuli were generated using MATLAB (The MathWorks, Natick, MA, USA) and the Psychophysics toolbox (Brainard, 1997), and presented on an LCD computer screen (BenQ XL2430T; 120 Hz refresh rate; 61 cm screen size in diameter; 1920 × 1080 pixels) with a grey background (15.1 cd/m^2^) and positioned approximately 70 cm from participants.

**Figure 2.**
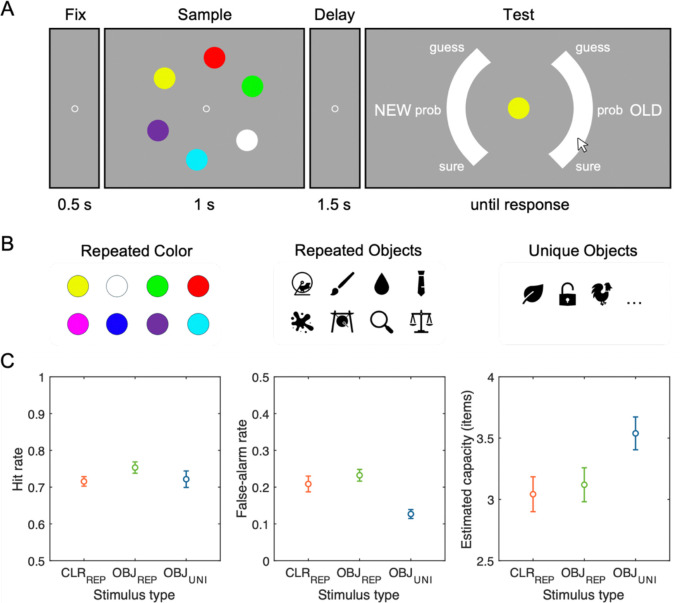
Experimental paradigm and behavioral results from Experiment 1. (A) Task sequence for the visual WM recognition task. Each trial began with a fixation display (500 ms), followed by a memory array containing six items (1,000 ms), a blank retention interval (1,500 ms), and a recognition probe requiring a confidence rating on a continuous scale ranging from “sure new” to “sure old”. (B) Examples of stimulus sets used across three conditions: colors (CLR_REP_; repeated across trials), meaningful objects repeated throughout the experiment (OBJ_REP_), and trial-unique meaningful objects presented only once (OBJ_UNI_). (C) Behavioral performance summarized as hit rate (left), false-alarm rate (middle), and estimated visual working memory capacity measured by Cowan’s *K* (right) for each stimulus condition. Error bars represent standard error of the means.

Figure 2 illustrates the stimuli and procedure of the task. Participants performed a visual WM recognition task with a set size of six items across three blocked conditions: trial-repeated colors, trial-repeated meaningful objects, and trial-unique meaningful objects. Block order was counterbalanced across participants. Each block contained 120 trials. Participants were given short breaks between blocks and 20-second breaks after every 40 trials within each block to reduce fatigue.

Each trial started with a 500 ms central fixation cross (0.2° visual angle), followed by a memory array of six items (each 2.0° × 2.0°) presented for 1,000 ms, arranged evenly along an imaginary circle (radius of 5.3°). After a 1,500 ms blank retention interval, participants saw a single central probe, with a semi-circular confidence rating scale (radius of 8.2°, thickness of 2.2°) split into left and right sides, separated by 90° gaps and with response regions labeled “NEW” and “OLD”, respectively. Participants responded by moving a mouse cursor from the screen center to the continuous scale labeled with discrete confidence markers, “surely-new”, “probably-new”, “guess-new”, “guess-old”, “probably-old”, and “surely-old”. Probe items were old (presented in the memory array) or new with equal probability.

In both trial-repeated conditions (colors and meaningful objects), memory arrays consisted of six items randomly selected from a fixed set of eight stimuli unique to each condition. The color set consisted of eight categorically distinct hues (RGB values: red [255,0,0], green [0,255,0], blue [0,0,255], magenta [255,0,255], yellow [255,255,0], cyan [0,255,255], orange [255,128,0], and white [255,255,255]). For objects, we selected Microsoft Office icons that were easily recognizable, including animals, plants, foods, transportation, musical instruments, and so forth (see Figure 2B for example stimuli). These items were presented in black on the grey background. The eight objects in the trial-repeated object condition were randomly sampled from the object pool for each participant to minimize potential influence of sampling bias in perceptual or semantic properties. The new probes were selected from the remaining items in the set of eight that were not presented in the current memory array. In contrast, for trial-unique objects condition, six novel objects were randomly selected each trial, with another previously unseen object used as the new probe.

#### Data Analysis

##### Behavioral Performance.

We calculated hit rates (correctly recognition of old probes) and false alarm rates (incorrect recognition of new probes as old) for each condition. From these, we computed Cowan’sK(capacity)=setsize×(hitrate-falsealarmrate).

##### Hierarchical Bayesian Dual-Process Signal Detection Modeling.

Confidence ratings were derived from the final clicking response along the semi-circular response regions and binned into six discrete confidence levels, specifically, three levels of confidence for “old” responses (surely-old, probably-old, and guess-old) and three levels for “new” responses (guess-new, probably-new, and surely-new). These ratings were used to construct receiver operating characteristic (ROC) curves, plotting cumulative false-alarm rates on the x-axis against cumulative and hit rates on the y-axis across confidence levels.

These empirical ROCs were fitted with the DPSD model. The DPSD model assumes that recognition decisions are based on two independent processes (Yonelinas et al., 2010; Yonelinas, 2002): 1) Recollection, which is an all-or-none retrieval of contextual details reflecting context-bound WM representation, and 2) Familiarity, which is a continuous signal-detection process reflecting context-free episodic LTM traces. The DPSD parameter estimation was performed using a hierarchical Bayesian method, which provides robust population-level estimates of the model parameters by simultaneously accounting for different sources of uncertainty across individual (random) and condition (fixed) effects. The advantage of the hierarchical Bayesian method is especially useful in ROC modeling, wherein a relatively limited number of trials are allocated for each decision criterion (e.g., six-binned confidence level) per experimental condition (Park et al., 2023). The main and interaction effects were estimated in a general linear model, sampled from the normal distribution where the mean is the sum of the fixed and random effects and the variability term is the interaction across effects.

**Figure 3.**
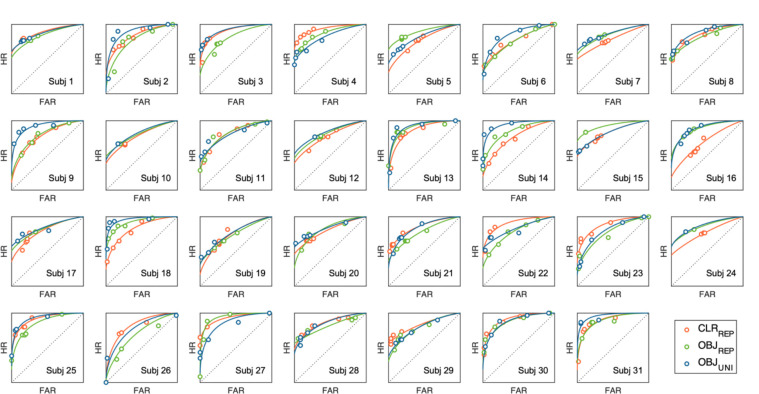
Observed and model-fitted ROC curves for individual participants. Each panel shows hit rate (HR) plotted over false-alarm rate (FAR) across confidence levels from each participant. Solid curves represent model predictions generated by the mean posterior parameters estimated from the hierarchical Bayesian dual-process signal detection model. Color codes represented repeated-colors (CLR_REP_ in red), repeated-objects (OBJ_REP_ in green), and unique-objects (OBJ_UNI_ in blue) conditions, respectively. Circles denote observed data. The close correspondence between predicted and observed values indicates good individual-level model fit.

We used Markov chain Monte Carlo sampling to estimate the posterior distributions of the parameters, generating 12,000 samples after 12,000 warm-up iterations. Population-level parameter posteriors were thus represented by a 12,000 × 31 × 3 matrix (i.e., samples × participants × stimulus types). We chose noninformative and reasonably informative priors. Model convergence was confirmed by using the Gelman–Rubin diagnostic $\hat{R}$ (Gelman & Rubin, 1992), and found to be close to or equal to 1.0 for all population-level parameters. Figure 3 provides visualization of model fits over empirical ROCs for individual participant. Statistical inference was made based on posterior mean parameter estimates and their associated 95% highest density intervals (HDI_95%_). HDIs provide direct estimates of evidence strength, intervals that exclude zero provide strong evidence for credible effects (Kruschke, 2015).

### Results

#### Behavioral Performance

Figure 2C summarizes the hit rates, false alarm rates, and Cowan’s *K* values across the three experimental conditions. First, a one-way repeated-measures analysis of variance (ANOVA) on the mean Cowan’s *K*s revealed a significant main effect of stimulus type, *F*(2, 90) = 3.74, *p* = .028, *η*^*2*^_*p*_ = .08. This effect was found to be primarily driven by higher capacity estimates for trial-unique objects (*M* = 3.54, *SD* = 0.75) compared to both trial-repeated colors (*M* = 3.04, *SD* = 0.79) and trial-repeated objects (*M* = 3.12, *SD* = 0.77), *ts*(30) > 2.69, Bonferroni-corrected *ps*_*bonf*_ < .036, *ds* > 0.49. Critically, Cowan’s *K* estimates did not significantly differ between trial-repeated colors and trial-repeated objects, *t*(30) = −0.42, *p*_*bonf*_ = 1.000, *d* = −0.08.

Further analysis of hit and false alarm rates revealed that the observed advantage for trial-unique objects primarily stemmed from its reduced false alarms rather than enhanced hit rates. Specifically, for hit rates, a one-way repeated-measures ANOVA indicated no significant effect of stimulus type, *F*(2, 90) = 1.36, *p* = .263, *η*^*2*^_*p*_ = .03, with highly comparable performance for trial-repeated colors (*M* = 0.72, *SD* = 0.07), trial-repeated objects (*M* = 0.75, *SD* = 0.09), and trial-unique objects (*M* = 0.72, *SD* = 0.13), *ts*(30) < 1.93, *ps*_*bonf*_ > .188, *ds* < 0.35. In contrast, false alarm rates varied significantly by stimulus type, *F*(2, 90) = 10.61, *p* < .001, *η*^*2*^_*p*_ = .19, with trial-unique objects showing substantially fewer false alarms (*M* = 0.13, *SD* = 0.07) compared to both trial-repeated colors (*M* = 0.21, *SD* = 0.12) and trial-repeated objects (*M* = 0.22, *SD* = 0.09), *ts*(30) > 3.85, *ps*_*bonf*_ < .002, *ds* > 0.70. False alarm rates did not differ significantly between trial-repeated colors and trial-repeated objects, *t*(30) = 1.06, *p*_*bonf*_ = .889, *d* = 0.19.

#### Dual-Process Signal Detection Modeling

**Figure 4.**
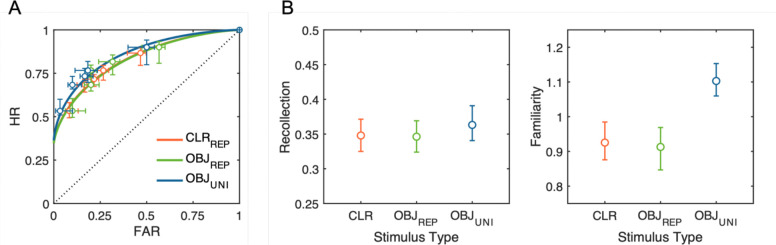
Observed and model-predicted ROC curves and posterior parameter estimates from the hierarchical Bayesian DPSD model. (A) ROC curves for each stimulus condition: repeated colors (CLR_REP_; red), repeated meaningful objects (OBJ_REP_; green), and trial-unique meaningful objects (OBJ_UNI_; blue). Circles represent observed mean hit rates (HR) and false-alarm rates (FAR) across confidence levels, and the horizontal and vertical error bars indicate standard errors of the mean FAR and HR, respectively. Solid lines depict model-predicted ROC curves based on posterior mean values of the model parameters. (B) Posterior means and 95% highest density intervals (HDIs_95%_) for the population-level recollection (left) and familiarity (right) parameters, across stimulus types. The boundaries of HDI_95%_ not crossing over between conditions indicate a statistically credible difference.

The hierarchical Bayesian DPSD model revealed a clear dissociation between recollection and familiarity processes across stimulus types (Figure 4). Recollection estimates were highly comparable across all three conditions, with substantial overlaps in their posterior distributions between trial-repeated colors (*M* = 0.35, *HDI*_*95%*_ [0.33, 0.37]), trial-repeated objects (*M* = 0.35, *HDI*_*95%*_ [0.32, 0.37]), and trial-unique objects (*M* = 0.36, *HDI*_*95%*_ [0.34, 0.39]). In contrast, familiarity estimates exhibited a robust effect consistent with the results of Cowan’s *K*. Familiarity was credibly higher for trial-unique objects (*M* = 1.10, *HDI*_*95%*_ [1.06, 1.15]) compared to both trial-repeated colors (*M* = 0.93, *HDI*_*95%*_ [0.88, 0.98]) and trial-repeated objects (*M* = 0.91, *HDI*_*95%*_ [0.85, 0.97]), with non-overlapping credible intervals providing strong evidence for this difference.

### Discussion

Experiment 1 demonstrated that improved memory performance for meaningful relative to simple objects may reflect reduced PI with trial-unique objects rather than increased WM capacity for meaningful items. Replicating past studies, we observed a clear performance advantage for meaningful objects, but only when the design employed trial-unique stimuli (Endress & Potter, 2014). This meaningful object benefit was eliminated when PI susceptibility was equated through repeated stimulus presentations. Further behavioral analyses revealed that the trial-unique object benefit was driven specifically by a reduction in false alarms rather than by enhanced hit rates. These findings indicate that the apparent advantage does not reflect expanded visual WM capacity per se, but rather the use of trial-unique stimuli that are less susceptible to PI.

Finally, hierarchical DPSD modeling of the empirical ROCs revealed a key dissociation between underlying recognition processes associated with the observed behavioral pattern. The recollection parameter, hypothesized to reflect context-bound WM representations, remained comparable across all stimulus type conditions, while familiarity reflecting context-free episodic LTM traces was selectively enhanced for trial-unique meaningful objects. Thus, the meaningful object advantage under trial-unique conditions appears predominantly to be attributed to enhanced familiarity signals provided by episodic LTM rather than expanded active WM storage capacity.

## Experiment 2

While the results of Experiment 1 suggest that the meaningful object advantage reflects reduced PI and enhanced contributions from LTM rather than expanded WM capacity, direct measurement of the number of neurally-active representations in WM provides a powerful complement to the behavioral evidence. Thus, Experiment 2 examined the CDA, a neural marker of active WM storage, in a lateralized change detection task involving trial-repeated colors and trial-unique meaningful objects across varying set sizes (1, 3, or 5 items).

**Figure 5.**
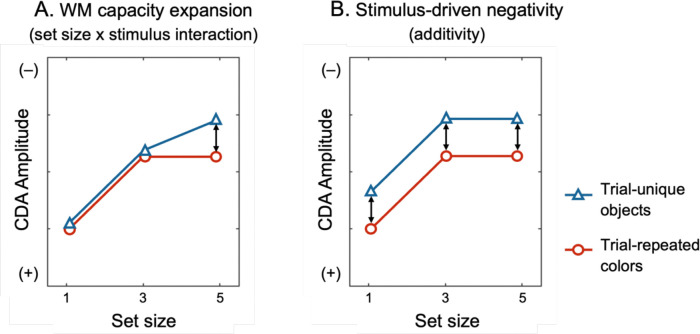
Hypothetical CDA amplitude patterns predicted by competing hypotheses. (A) If remembering meaningful objects truly expands visual WM capacity, the CDA amplitude should exhibit a significant set size × stimulus type interaction effect, with CDA amplitude increasing across a larger range of set sizes (set size 5). (B) By contrast, a stimulus-related confound hypothesis predicts only additive amplitude differences between stimulus types across all set sizes, without interaction.

A critical prediction centers on the interaction between set size and stimulus type (Figure 5). If meaningful objects increase visual WM capacity, CDA amplitude should rise with set size and plateau at a higher level for meaningful objects than for simple features, producing a significant set size × stimulus type interaction. Alternatively, if the advantage reflects contributions from episodic familiarity under PI-free conditions, no change in the shape of the CDA by set size function is predicted. Importantly, by measuring CDA activity across multiple set sizes, we will be able to tell whether any observed differences in CDA activity reflect differences in WM storage (yielding an interaction between stimulus type and set size) or stimulus-driven effects that are independent of changes in the number of items stored in WM.

### Method

#### Participants

Twenty-five volunteers (14 female) participated in Experiment 2. All participants were right-handed, aged 19–32 years (*M* = 23.7, *SD* = 3.1), and reported normal or corrected-to-normal vision. Informed consent was obtained according to procedures approved by the University of Chicago Institutional Review Board. The final sample size of 18 participants and number of trials collected was determined based on prior EEG studies investigating CDA amplitude differences under various stimulus conditions, with an eye towards studies examining the CDA amplitude by set size function (Fukuda et al., 2015; Luria et al., 2016; Woodman & Vogel, 2008).

#### Stimuli and Procedure

**Figure 6.**
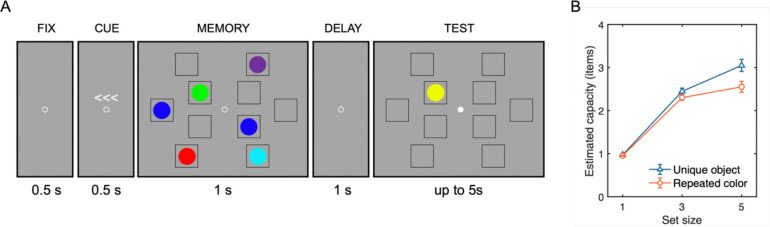
Procedure and resulting capacity estimates for Experiment 2. (A) Lateralized visual WM task sequence shown for the color stimulus condition. Each trial began with central fixation (500 ms), followed by an arrow cue indicating the task-relevant hemifield (left or right; 500 ms). The memory array was then presented laterally (1,000 ms), followed by a blank retention interval (1,500 ms). Participants subsequently indicated whether a test probe matched the remembered item at the corresponding location. The fixation point changed its color to white to indicate the onset of test array and allow participants to blink. The trial-unique meaningful object condition used the same procedure, differing only in stimulus content. (B) Capacity estimates of mean Cowan’s *K*, as a function of set size (1, 3, or 5 items) and stimulus type (unique object vs. repeated color). Error bars represent standard error of the mean.

Participants completed a lateralized change detection task in two blocked conditions, trial-repeated colors and trial-unique objects, respectively (Figure 6A). Stimulus generation was identical to Experiment 1. Each trial began with a 500 ms central fixation (radius of 0.2°), followed by a 500 ms arrow cue presented above the fixation circle, indicating the side to remember (left/right, equally probable). A memory array was presented for 1,000 ms. In order to account for the lateralized nature of the CDA component, the memory array contained 1, 3, or 5 items presented in each visual hemifield. For both hemifields, the matching number of items were displayed at randomly chosen locations among five fixed placeholders (3.0° × 3.0° each), two were on an inner imaginary circle of 2.7° radius and the other three were on an outer imaginary circle of 7.0° radius, with 2.0° lateralized offset from the central midline of the display. After a 1000 ms retention interval displaying only placeholders, a test probe appeared at one of the remembered item locations on the cued side. Participants indicated whether the probe matched the original item by pressing one of two buttons, “z” or “/” key to indicate “no-change” or “change”, respectively. The probability of change and no-change was equal. Participants completed 200 trials per set size condition in each of the repeated-color and unique-object blocks with a randomized block order across participants.

#### EEG Acquisition

EEG was recorded from 30 active Ag/AgCl electrodes (actiCHamp, Brain Products, Munich, Germany) mounted in an elastic cap positioned according to the international 10–20 system (Fp1, Fp2, F7, F3, F4, F8, Fz, FC5, FC6, FC1, FC2, C3, C4, Cz, CP5, CP6, CP1, CP2, P7, P8, P3, P4, Pz, PO7, PO8, PO3, PO4, O1, O2, Oz). A ground electrode was placed at position Fpz and two additional electrodes were affixed with stickers to the left and right mastoids. Data were referenced online to the right mastoid and re-referenced offline to the algebraic average of the left and right mastoids. Incoming data were filtered (low cutoff = 0.01 Hz, high cutoff = 80 Hz; slope from low to high cutoff = 12 dB/octave) and recorded with a 500 Hz sampling rate. Impedance values were kept below 10 kΩ.

Eye movements and blinks were monitored using electrooculogram (EOG) activity and eye tracking. We collected EOG data with five passive electrodes (two vertical VEOG electrodes placed above and below the right eye, two horizontal HEOG electrodes placed ~1 cm from the outer canthi of each eye, and a ground electrode placed on the left cheek). Eye-tracking data was collected using a desk-mounted EyeLink 1000 Plus eye-tracking camera (SR Research, Ontario, Canada) sampling at 1,000 Hz.

#### Artifact Rejection

EEG data were segmented into epochs (−200 to 2,000 ms from memory array onset). An automatic artifact rejection pipeline was applied to detect eye movements, blinks, and EEG artifacts. We implemented a comprehensive set of criteria using ERPLAB functions (Lopez-Calderon & Luck, 2014). Trials contaminated by artifacts were excluded for EEG analyses but not from behavioral analyses. We rejected trials containing eye movements and blinks using EOG channels and eye-tracking data.

**Figure 7.**
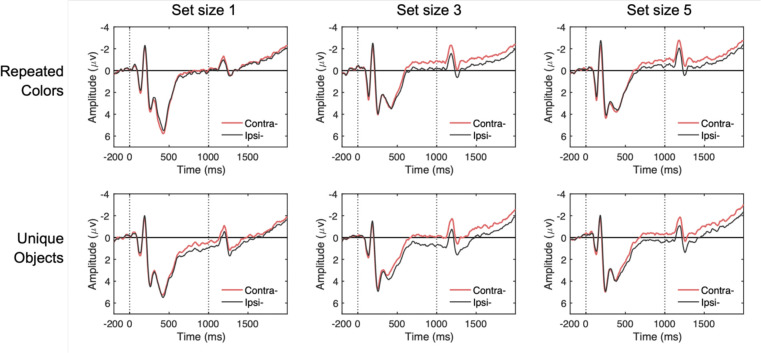
Grand-averaged contralateral and ipsilateral waveforms by stimulus type and set size. Waveforms are time-locked to the memory array onset (0 ms) and offset (1000 ms) and averaged across posterior electrode sites (P3/P4, P7/P8, PO3/PO4, PO7/PO8) in Experiment 2. Each panel compares contralateral (red) and ipsilateral (black) activity for trial-repeated colors (top row) versus trial-unique objects (bottom row) at set sizes 1, 3, and 5 (left to right). Vertical dotted lines mark the onset and offset of the memory array.

For EOG channels, trials were flagged when the absolute voltage exceeded 50 μV or when step-like activity exceeded 30 μV within a 100 ms moving window (advanced in 10 ms steps). For eye-tracking rejection, we applied a similar sliding window to the x-gaze coordinates and y-gaze coordinates (window size = 100 ms, step size = 10 ms, threshold = 0.5°). When eye-tracking data were not available, we used EOGs to detect saccades and blinks. For EEG artifacts, we checked for drift (e.g., skin potentials) using the *pop_rejtrend* function, excluding trials in which a line fitted to the EEG data had a slope exceeding 75 μV with a minimal *R*^*2*^ of 0.3. High-frequency noise and muscle artifacts were detected using the *pop_artmwppth* function, excluding trials with peak-to-peak activity greater than 75 μV within a 200-ms sliding window advanced in 100-ms steps. We also excluded trials with absolute voltage exceeding ±100 μV in any EEG channel using the *pop_artextval* function. Additionally, we detected step-like artifacts (which can occur with electrode movement) using the *pop_artstep* function, flagging trials with voltage changes exceeding 60 μV within a 150-ms sliding window advanced in 10-ms steps. For ocular artifacts, we applied separate criteria to EOG channels, using an absolute voltage threshold of ±50 μV and detecting saccade-like step functions exceeding 30 μV within a 100-ms sliding window advanced in 10-ms steps. Trials containing flatline signals were also excluded. Seven participants with rejection rate greater than 30% of trials were excluded from further analysis. Across the remaining participants, an average of 12.7% of trials were rejected, with no significant differences in rejection rates between stimulus conditions or set sizes. CDA was calculated as mean amplitude differences between contralateral and ipsilateral posterior electrodes (PO3/PO4, PO7/PO8, P3/P4, P7/P8; Figure 7). The average CDA amplitudes were taken from three different measurement windows, during stimulus presentation (i.e., encoding; 400–1,000 ms), delay period (1,400–2,000 ms), and a combined window of 400–2,000 ms following memory onset.

### Results

#### Behavioral Performance

Behavioral results mirrored those of Experiment 1 (Figure 6B). A two-way repeated-measures ANOVA on Cowan’s *K* as a function of stimulus type (trial-repeated color vs. trial-unique object) and set size (1, 3, vs. 5 items) revealed significant main effects of stimulus type, *F*(1, 24) = 13.61, *p* = .001, *η*^*2*^_*p*_ = .36, and set size, *F*(2, 48) = 46.28, *p* < .001, *η*^*2*^_*p*_ = .91. The set size × stimulus type interaction effect was also significant, *F*(2, 48) = 12.76, *p* < .001, *η*^*2*^_*p*_ = .35. Notably, the interaction effect was primarily driven by a significant difference in the *K* estimate at set size 5, with trial-unique objects being higher capacity estimates (*M* = 3.05, *SD* = 0.70) compared to trial-repeated colors (*M* = 2.55, *SD* = 0.63), *t*(24) = 4.04, *p*_*bonf*_ = .001, *d* = 0.82. The difference in the *K* estimates between stimulus type at set size 1 (for unique objects: *M* = 0.97, *SD* = 0.04; for repeated colors: *M* = 0.95, *SD* = 0.04) and set size 3 (for unique objects: *M* = 2.45, *SD* = 0.37; for repeated colors: *M* = 2.30, *SD* = 0.33) did not yield statistical significance, *ts*(24) < 2.45, *ps*_*bonf*_ > .066, *d* < 0.50.

#### CDA Results

**Figure 8.**
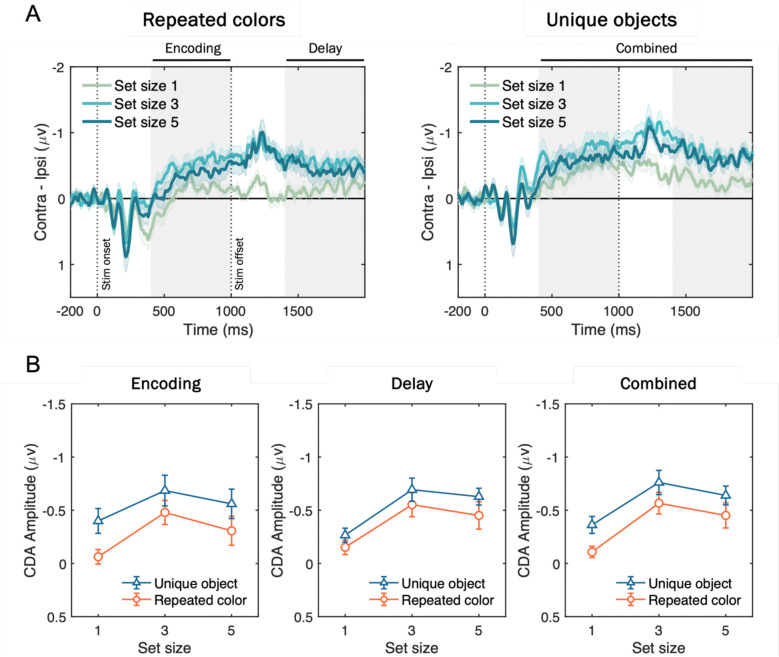
Grand-averaged CDA waveforms and mean amplitudes for trial-repeated colors and trial-unique objects. (A) CDA activity (contralateral minus ipsilateral activity at posterior electrode sites; P3/P4, P7/P8, PO3/PO4, PO7/PO8) time-locked to the memory onset and averaged across participants, plotted separately for repeated colors (left) and unique objects (right) at set sizes 1 (green), 3 (sky blue), and 5 (blue). Vertical dotted lines mark stimulus onset (0 ms) and offset (1000 ms), and gray shading indicates Encoding (400–1000 ms from stimulus onset), Delay (1400–2000 ms), and Combined (400–2000 ms) measurement windows. Shaded error bars represent standard error of the means (SEM). (B) Mean CDA amplitudes during the early (left), late (middle), and combined (right) time windows as a function of set size for unique objects (triangles on blue line) versus repeated colors (circles on red line). Error bars represent SEM.

Figure 8 shows the grand-averaged CDA waveforms and mean amplitudes across different time windows. In the combined analysis window (Figure 8B, right), a two-way repeated-measures ANOVA with factors of stimulus type (trial-repeated color vs. trial-unique object) and set size (1, 3, 5 items) revealed significant main effects of stimulus type, *F*(1, 17) = 21.33, *p* < .001, *η*^*2*^_*p*_ = .56, and set size, *F*(2, 34) = 19.42, *p* < .001, *η*^*2*^_*p*_ = .53. Critically, the set size × stimulus type interaction was not significant, *F*(2, 34) = 0.28, *p* = .760, *η*^*2*^_*p*_ = .02, indicating that although meaningful objects elicited larger CDA amplitudes overall, this effect was *additive* with set size, indicating that it reflected a *stimulus-driven* increase in contralateral negativity that was independent of the number of items stored in WM.

These results patterns remained the same when analyzing the separate time windows during stimulus presentation (i.e., encoding; Figure 8B, left) or delay period (i.e., delay; Figure 8B, middle), both showing that the two-way interaction effects did not achieve statistical significance, *F*(2, 34) = 1.44, *p* = .250, *η*^*2*^_*p*_ = .08 (encoding), and *F*(2, 34) = 0.01, *p* = .909, *η*^*2*^_*p*_ = .01 (delay). Furthermore, including measurement window as a factor (encoding vs. delay) in a three-way ANOVA revealed no significant main effect of window, *F*(1, 17) = 0.17, *p* = .682, *η*^*2*^_*p*_ = .01, nor a three-way interaction, *F*(2, 34) = 0.171, *p* = .843, *η*^*2*^_*p*_ = .01. these findings demonstrate that while meaningful objects elicited larger CDA amplitudes than colored squares, the effect was purely additive and did not reflect an increase in active storage capacity.

### Discussion

Using an electrophysiological measure of neurally active storage in WM, Experiment 2 provided evidence against the claim that more meaningful than simple objects can be stored in visual WM. Although behavioral data showed superior performance for trial-unique meaningful objects over repeated colors, the shape of the CDA by set size function revealed that the same number of items were stored in WM. If subjects were able to store more meaningful objects than simple colors, the difference in CDA amplitudes should have been larger for set size 5 where the clearest behavioral benefits were observed for meaningful objects over repeated colors. Instead, CDA differences between meaningful and simple objects were equivalent across all set sizes, suggesting a stimulus-driven contralateral negativity that is independent of the number of items stored in WM (Drew et al., 2011; Woodman & Vogel, 2008). Thus, online neural measures of WM storage disconfirm the hypothesis that WM capacity is expanded for meaningful objects.

## General Discussion

We present behavioral and neural evidence that calls into question recent claims that WM capacity is expanded for meaningful compared to simple objects. In Experiment 1, we independently manipulated meaningfulness and PI, and found that the performance advantage for meaningful stimuli was driven by trial-unique stimulus presentations that minimized PI rather than by meaningfulness, *per se*. When PI was equated by using repeated meaningful stimuli, capacity estimates for meaningful and simple stimuli were equated. Hierarchical Bayesian DPSD modeling further revealed that the “trial-unique advantage” specifically reflected enhanced familiarity signals from episodic LTM, while recollection-based WM processes remained stable across stimulus types. Thus, enhanced performance under trial-unique conditions can be explained by increased LTM contributions when PI is minimized, rather than by increased WM storage capacity.

Experiment 2 provided converging electrophysiological evidence using the CDA, a neural marker of active WM maintenance. Prior studies have reported higher CDA amplitudes for meaningful objects compared to simple features. However, those studies typically employed only a single set size, a design that cannot distinguish between changes in the number of items stored, and stimulus-driven changes in CDA amplitude that are independent of WM load. If meaningfulness truly increases the number of items stored in WM, CDA amplitude should exhibit an interaction between stimulus type and set size, exhibiting steeper amplitude increases across higher set sizes. Instead, the effect of meaningfulness was on CDA activity was additive with the number of items to be stored, indicating a stimulus-driven effect that is separate from changes in WM storage (Drew et al., 2011; Woodman & Vogel, 2008).

From a broader theoretical perspective, these findings align closely with embedded-process models of WM (Cowan, 2001; Oberauer, 2002), which posit a capacity-limited WM system collaborating constantly with a capacity-unlimited LTM system. According to these models, WM consists of a subset of activated LTM representations held in an immediately accessible state by attentional pointers bound to spatiotemporal contexts (Awh & Vogel, 2024; Oberauer & Lin, 2017). From this perspective, trial-unique meaningful stimuli enhance recognition performance by enabling greater reliance on global familiarity signals from LTM when PI is minimized, rather than by expanding WM capacity. In other words, performance benefits observed with meaningful stimuli under trial-unique thus PI-free conditions reflect leveraging of episodic familiarity signals, in the absence of any changes in fundamental WM capacity limits.

Our study highlights PI as a critical gating mechanism that regulates interactions between episodic LTM and active WM. Under low-PI conditions (e.g., trial-unique stimuli), episodic LTM provides reliable context-free familiarity signals that supplement recognition performance and enhance behavioral performance. Conversely, high-PI conditions (e.g., repeated stimuli) make episodic familiarity an unreliable marker of “oldness”, forcing greater reliance on context-specific recollection of active WM representations. This gating function clarifies why traditional WM paradigms with repeated stimuli yield stable capacity estimates, while trial-unique paradigms capacity estimates that can be an order of magnitude larger (Endress & Potter, 2014).

Furthermore, our findings contribute to evidence supporting hierarchical interactions between memory systems, comprising perceptual processes and contextual binding mechanisms (Christophel et al., 2017; Lee et al., 2023; Yatziv & Kessler, 2018). Perceptual memory provides large-capacity but interference-prone representations automatically encoded into activated LTM, whereas active WM entails selective binding mechanisms to ensure spatiotemporal tracking of individuated items. Recent neuroimaging and neurophysiological studies support this dual-code perspective, revealing distinct neural substrates for sensory-based perceptual memories maintained in posterior cortices versus abstract, context-bound representations supported by frontoparietal networks (Chunharas et al., 2024; Kwak & Curtis, 2022; Xu & Chun, 2006). Intracranial recordings similarly dissociate medial temporal neurons encoding detailed sensory and semantic features from frontal neurons associated with abstract, slot-like WM capacities (Kamiński et al., 2017).

Several limitations of the present study suggest promising directions for future research. For example, while we controlled PI through stimulus repetition, we did not systematically manipulate graded levels of PI or examine its spatial specificity (Endress, 2022; Donenfeld et al., 2024; Lin & Luck, 2012; Makovski, 2016). Investigating the temporal dynamics of PI build-up and release (Keppel & Underwood, 1962; Watkins & Watkins, 1975) could further clarify how global familiarity-based LTM processes can shape memory performance in WM procedures. Moreover, our findings should be considered in light of growing research on memorability, a stimulus-intrinsic property that makes certain images consistently better remembered across individuals (Bainbridge, 2019; Isola et al., 2014). Recent work shows that intrinsic memorability effects emerge during visual WM tasks (Tam et al., 2025; Ye et al., 2024). Future studies exploring interactions between PI and memorability may elucidate how stimulus properties and task contexts jointly influence memory performance, potentially integrating stimulus-centered and process-based accounts.

In conclusion, our comprehensive behavioral and neural data demonstrate that previously reported WM capacity advantages for meaningful objects primarily reflect reduced PI rather than genuine expansions of active storage capacity. These findings reinforce theoretical frameworks positing a fixed-capacity attentional pointer mechanism underlying active WM, and highlight PI as a critical gating factor regulating episodic LTM contributions to short-term memory task performance (Bartsch et al., 2024; Oberauer & Bartsch, 2023).

## Figures and Tables

**Figure 1.**
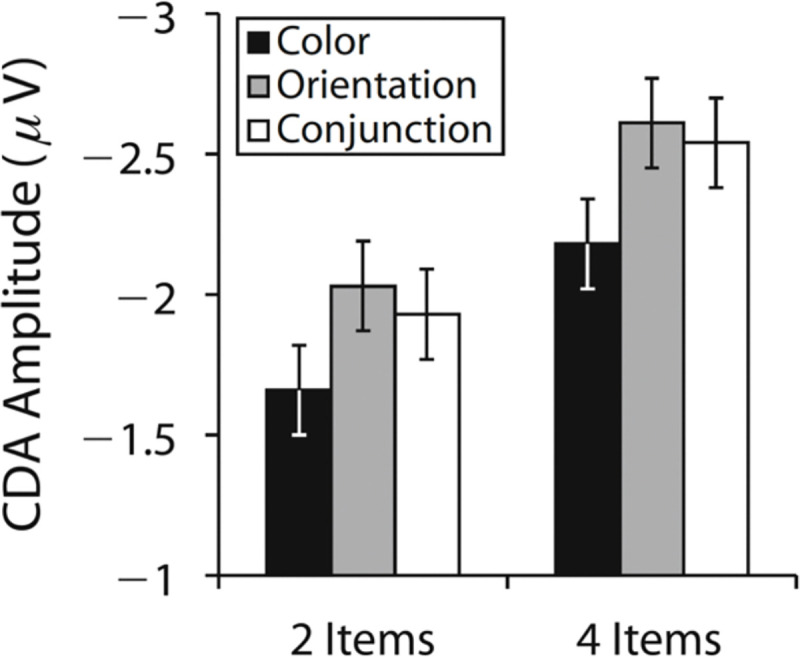
Mean contralateral delay activity (CDA) amplitudes for color, orientation, and conjunction stimuli at set sizes 2 and 4, adapted from Figure 3D of Woodman and Vogel (2008). Although orientation and conjunction stimuli elicited larger CDA amplitudes than color stimuli, these differences were additive across set sizes, suggesting that the enhanced CDA was not due to an increased number of items stored in working memory.

## Data Availability

All data generated or analyzed during this study are available via the Open Science Framework repository at https://osf.io/e8ptm/

## Footnotes

1 Even if a study employed a continuous ring of colors in a recall procedure, it is well known that observers perceive a small set of discrete categories within this color space (e.g., Bae et al., 2015)

2 The one exception to the use of a single set size is the study by Brady and colleagues (2016). However, there has been continued debate about the precise empirical pattern, because subsequent replication attempts have not been successful. Quirk and colleagues (2020) ran a replication study using the same stimuli and task, but did not observe CDA evidence for increased storage of meaningful objects. Moreover, Brady and colleagues (2016) reported that performance with meaningful objects benefitted more from increased exposure durations than did simple colors, but this effect was not replicated either. Given uncertainty regarding the robustness of these findings, we focus our discussion on the other published studies that examine CDA activity with meaningful and simple objects.
